# Comparative genomics reveal signatures of ecological specialization in the striped ambrosia beetle *Trypodendron lineatum*

**DOI:** 10.1186/s12864-026-13049-3

**Published:** 2026-06-16

**Authors:** Zaide Montes-Ortiz, Daniel Powell, Heiko Vogel, Christer Löfstedt, Martin N. Andersson

**Affiliations:** 1 Department of Biology, Lund University, Kontaktvägen 10, Lund, SE-223 62 Sweden; 2 Max Planck Center next Generation Insect Chemical Ecology (nGICE), Lund University, Lund, Sweden; 3 Centre for Bioinnovation, University of the Sunshine Coast, Sippy Downs, Queensland Australia; 4 Department of Insect Symbiosis, Max Planck Institute for Chemical Ecology, Jena, Germany

**Keywords:** Coleoptera, Curculionidae, Scolytinae, Symbiosis, Genome annotation, Gene family evolution, Immune gene contraction, Detoxification

## Abstract

**Background:**

Beetles (Coleoptera) display exceptional dietary diversity and occupy a wide range of ecological niches, often involving close associations with plants and microbes. Ambrosia beetles (Curculionidae; Scolytinae and Platypodinae) exemplify ecological specialization by cultivating mutualistic fungi within galleries excavated in their host trees’ xylem, with the fungi serving as their main food source. The striped ambrosia beetle *Trypodendron lineatum* is a pest of conifers, relying on its nutritional mutualist *Phialophoropsis ferruginea* for survival. This fungiculture-based lifestyle provides a system for exploring how specialized mutualism is reflected at the genomic level. Hence, we performed a comparative genomics analysis between *T. lineatum* and nine other beetle species with different ecological specializations. We hypothesized that fungiculture is associated with specific genomic adaptations, including changes in gene family composition related to nutrition, detoxification, and immunity.

**Results:**

The small genome of *T. lineatum* (74.4–83.6 Mb) exhibits comparatively low levels of repetitive DNA (19.9%), including a reduced proportion of transposable elements. Annotation generated 14,830 high-quality gene predictions, most of which were supported by transcript evidence or functional domains. Comparative orthology analysis across ten beetle species identified 13,896 orthogroups, with *T.lineatum* having 78 species-specific orthogroups comprising 238 genes. Gene family evolution analyses revealed 33 families with significant size changes in *T. lineatum*, including 16 expansions and 17 contractions. Notably, gene families associated with digestion, detoxification, and immunity were contracted. These included glycoside hydrolase 28, cytochrome P450, serpin, and trypsin families, which may reflect the fungus-based, rather than plant-based, diet of *T. lineatum*, and reduced reliance on broad-spectrum immune defenses. In contrast, expansions in the THAP and CD80-like immunoglobulin domain families indicate diversification of genes involved in genomic regulation and immune recognition.

**Conclusions:**

Our results suggest that the genome of *T. lineatum* is characterized by low repeat content and compact gene architecture. The observed contractions in key gene families involved in plant digestion, detoxification, and immunity may represent genomic signatures of its obligate mutualistic specialization and narrow ecological niche. Our findings provide the first insights into the genomic adaptations of fungus-farming ambrosia beetles, suggesting that co-evolved insect-microbe mutualisms may lead to reductions in a variety of gene families.

**Supplementary Information:**

The online version contains supplementary material available at 10.1186/s12864-026-13049-3.

## Introduction

Beetles (Coleoptera) represent one of the most species-rich and ecologically diverse lineages in the animal kingdom, comprising over 380,000 described species across a wide range of terrestrial and freshwater ecosystems [1, 2]. Moreover, their evolutionary success has been attributed in part to their remarkable diversity of feeding strategies, allowing them to exploit a wide variety of ecological niches [3, 4]. These include phytophagy (feeding on plants), saproxyly (feeding on decaying wood) [5], predation [6], and mycophagy (fungus-feeding) [7, 8]. Importantly, several of these feeding strategies are often accompanied by symbiotic relationships with microbes [9]. This dietary versatility has not only fostered extensive diversification but has also driven functional innovations at morphological (e.g., specialized mouthparts) [10], physiological (e.g., digestive enzymes) [11, 12], and genomic (e.g., detoxification-related and chemosensory genes) levels [13–16].

Bark- and ambrosia beetles (family Curculionidae) are both members of the subfamily Scolytinae. However, ambrosia beetles are a polyphyletic group with members also in Platypodinae, and they differ from bark beetles in terms of feeding behavior, larval development, and associations with symbiotic microbes [17, 18]. Bark beetles primarily feed on the host tree phloem inoculated with beetle-associated symbiotic fungi. Inside the phloem, their larvae construct individual tunnels away from the maternal gallery, with limited parent-offspring interaction [7, 17–19]. In contrast, ambrosia beetles bore into the xylem and cultivate obligate mutualistic fungi within their galleries. Rather than feeding on wood directly, they rely on these fungal gardens as their sole nutritional source [20–22]. This tight interdependence exemplifies a co-evolved obligate nutritional mutualism that has shaped the ecological strategies and life histories of ambrosia beetles in fundamentally different ways from their bark beetle relatives [7]. This symbiotic relationship is maintained through complex mechanisms, including the ability of ambrosia beetles to store and maintain fungal propagules (including spores and mycelial fragments) in a dormant state within specialized glandular mycangia. This process has likely had a significant evolutionary impact on, for example, their immune system and associated genes [23], favoring adaptations that mitigate immune responses against the fungi.

The striped ambrosia beetle, *Trypodendron lineatum* (Olivier), is a significant pest species of Holarctic conifer forests [20, 24, 25]. It targets stressed or dying conifer trees, boring into the xylem to establish larval galleries, which are inoculated with spores of its obligate fungal mutualist, *Phialophoropsis ferruginea* (Mathiesen-Käärik) (Ascomycota) [26]. This fungus-farming lifestyle is a prime example of niche construction in which *T. lineatum* actively modifies its environment effectively [27, 28]. By inoculating host trees with its fungal mutualist and maintaining the growth of fungal gardens within the galleries, *T. lineatum* chemically and structurally alters the xylem environment, creating a stable and nutritionally optimized niche for larval development, thus enhancing survival and reproductive success.

To find suitable host trees for colonization, volatile organic compounds from decaying conifers, such as α-pinene and ethanol [29], attract *T. lineatum*, whereas volatiles from non-host angiosperm plants provide cues that help discriminate against unsuitable hosts [13, 20].The beetle also uses the female-produced aggregation pheromone (+)-lineatin (3,3,7-trimethyl-2,9-dioxatricyclononane) [30], which attracts both sexes to host trees. Moreover, the presence of olfactory sensory neurons that specifically respond to volatiles produced by its fungal mutualist highlights a finely tuned sensory system for locating suitable habitats and maintaining the fungal association [13]. Hence, the strong dependence of *T. lineatum* on both host trees and fungal partners makes it an ideal model for studying the genomic consequences of mutualistic specialization, especially in comparison with closely related bark beetles (Curculionidae: Scolytinae) that exhibit distinct ecological strategies and host associations [31–33]. For example, previous genomic studies have reported expansions of plant cell wall–degrading enzymes (PCWDEs) and reduced cytochrome P450 repertoires in *Ips typographus* L. (Eurasian spruce bark beetle) [31], diversified detoxification and cell wall–degrading gene families in *Dendroctonus ponderosae* Hopkins (mountain pine beetle) [33, 34], and compact chemosensory and detoxification gene sets in *Hypothenemus hampei* Ferrari (coffee berry borer) [32, 35]. These contrasting patterns provide a comparative framework for investigating how obligate fungal mutualism may shape gene family evolution in *T. lineatum*. To the best of our knowledge, such studies have never been performed on an ambrosia beetle. In fact, *T. lineatum* is one of the few ambrosia beetle species with a sequenced genome [36], making this species a prime target for such analysis.

Ecological specialization, particularly in species that engage in obligate mutualisms, is often accompanied by genomic signatures that reflect adaptation to narrow niches [3, 37]. A previous study showed that *T. lineatum* possesses a reduced repertoire of chemoreceptor-coding genes compared to other scolytine beetles, potentially reflecting a streamlined sensory system tailored to its specific associations with its host trees and fungal mutualist [36]. However, it remains unclear whether such specialization has had broader impacts on other gene families involved in e.g. digestion, detoxification, immunity, or development. To address this, we performed a comparative genomic and orthology analysis of *T. lineatum* and nine other beetle species, including related scolytine bark beetles, as well as outgroup species from other coleopteran families. The species included both closely related Curculionidae species, enabling comparisons within a shared evolutionary framework, alongside species from more distantly related families, representing diverse feeding habits and lifestyles, including fungivory, phloem-feeding, herbivory, xylophagy, and saprophagy (*Additional File 1*,* Supplementary Table 1*). We focused on the expansion and contraction of gene families as a proxy for functional adaptation, with the aim of uncovering how ecological constraints and mutualistic strategies have shaped the genome of *T. lineatum*.

## Methods

### Data sources

The *Trypodendron lineatum* genome assembly used in this study was generated and published previously [36], and it was retrieved from the European Nucleotide Archive (ENA; EMBL-EBI accession PRJEB74033; accessed January 2024). Additionally, three distinct datasets were included in this study: (1) a set of target beetle genomes, (2) annotated proteins retrieved from public databases, and (3) whole-body transcriptomes of *T. lineatum* generated in the present study as well as an antennae transcriptome from [36] (NCBI accession number PRJNA1126204; https://www.ncbi.nlm.nih.gov/sra/PRJNA1126204). In brief, for (1), we downloaded 13 additional beetle genomes from different families (accessed: January 2024) deposited in the database *GenBank* from the National Center for Biotechnology Information (NCBI); accession numbers for each species can be found in *Additional File 1*,* Supplementary Table 1*. For (2), we downloaded a non-redundant, well-annotated set of proteins from the *RefSeq* by using the query “(“beetle species“[Organism] OR beetle species [All Fields]) AND refseq[filter],”. This query was applied specifically to the ten beetle species selected for orthology inference (see below), whereas the additional beetle genomes were used only for genome quality comparisons and contextual analyses. Protein data for *I. typographus*,* H. hampei*,* and Callosobruchus maculatus* were obtained from public repositories (*Additional File 1*,* Supplementary Table 1*). Target beetle genomes (1) were selected based on their taxonomic relationship to *T. lineatum*, representation of diverse feeding habits and ecological strategies, and the availability of publicly available annotated genome assemblies.

### Genome size estimation and search for telomeres

To confirm and contextualize previously published assembly characteristics, the genome size of *T. lineatum* was, in the present study, estimated using a k-mer-based approach. Raw sequencing reads were first processed with Trimmomatic (v0.36) for quality control [38]. K-mer frequency analysis was then performed using Jellyfish (v2.2.10) with a k-mer length of 21, and the resulting distribution was analyzed by GenomeScope (v2.0) to determine genome size, heterozygosity, and repeat content [39, 40]. To contextualize the estimated genome size, we used QUAST (v.5.3.0) to compare it with the genome assemblies of 13 other beetle species [41]. Furthermore, telomeric regions within the *T. lineatum* genome were searched for using the Telomere Identification toolKit (tidk) [42] and the FindTelomer.py script (available at https://github.com/JanaSperschneider/FindTelomeres). We specifically searched for the telomeric repeat motifs TTAGG, TTAGGG, CCCTAA, and TCAGG commonly found in Coleoptera [43].

### Transcriptome sequencing, read mapping, and assembly

Adult *T. lineatum* males and females for transcriptome sequencing were collected from a conifer dominated forest in Tågaröd, South Sweden, during May 2023 using pheromone-baited (Lineatin Kombi dispensers, Witasek, Austria) traps (WitaTrap, 12 funnel size, Witasek). The whole bodies of six females and six males (in two separate samples) were used to isolate total RNA using the RNeasy Minikit (Qiagen, Hilden, Germany), according to the manufacturer’s instructions. Then, the RNA was DNAse-treated and subjected to library construction using the Illumina TrueSeq stranded mRNA (polyA) kit (Illumina, San Diego, CA, USA). The samples were sequenced on a NovaSeq6000 (NovaSeq Control Software 1.8.1/RTA v3.4.4) with a 151nt(Read1)-19nt(Index1)-10nt(Index2)-151nt(Read2) setup using ‘NovaSeqXp’ workflow in ‘S4’ mode flowcell. The raw reads were initially assessed for quality using FastQC (v2.2.1). Adapter trimming and quality filtering were performed using Trimmomatic [38], followed by a secondary quality assessment with FastQC (v2.2.1) [44], with summary reports generated by MultiQC [45]. High-quality reads were mapped to the *T. lineatum* reference genome using HISAT2 (v2.2.1) [46]. *De novo* transcriptome assembly was conducted with Trinity (v2.9.1) [47]. Additionally, CD-HIT-EST was performed with a sequence identity threshold of 0.98 to reduce redundancy among the assembled transcripts [48]. Finally, coding regions within the assembled transcripts were predicted using TransDecoder (v5.7.0) (available at https://github.com/TransDecoder/TransDecoder).

### Genome annotation and quality assessment

The genome of *T. lineatum* [36] was annotated using two complementary pipelines: MAKER (v3.01.2) [49] and BRAKER (v3) [50]. Before annotation, repetitive elements were identified by constructing an ad hoc repeat library directly from the *T. lineatum* genome assembly using RepeatModeler (v4.1.0) [51]. This custom library was combined with the comprehensive Repbase repeat database and subsequently applied with RepeatMasker (v4.0.8) to soft-mask repetitive regions in the genome [52]. Following the masking step, the MAKER annotation pipeline was executed in four iterative rounds. Annotation quality was assessed after each annotation round by calculating the Annotation Edit Distance (AED) distribution, which provides a quantitative measure of agreement between gene models and available evidence [53]. In the initial round, the annotation process incorporated various sources of evidence data, including transcriptome assemblies derived from whole-body and antennal tissues of *T. lineatum*, as well as the predicted protein sets from nine coleopteran species (*D. ponderosae*, *Dendroctonus valens* (LeConte), *H. hampei*, *I. typographus* (all Curculionidae), *Aethina tumida*, *Anoplophora glabripennis* (Motschulsky) (Cerambycidae), *Diabrotica virgifera* LeConte, *Leptinotarsa decemlineata* (Say) (both Chrysomelidae), *Nicrophorus vespilloides* (Herbst) (Silphidae), *Tribolium castaneum* (Herbst) (Tenebrionidae) and *Drosophila melanogaster* Meigen (Diptera) (*Additional File 1*,* Supplementary Table 1*). Additionally, the Insecta_Odb10 database (available at https://busco.ezlab.org/), containing 1,367 core genes representing 75 insect species across 14 orders, was included to enhance the accuracy and completeness of the first gene model prediction.

In the second round, the gene models generated in the initial round were used as input to train gene prediction software, specifically AUGUSTUS (v3.4.0) [54] with BUSCO (v5.2.2) [55] and SNAP (v1) [56]. The outputs from these trained predictors were then integrated into MAKER to generate refined second-round gene models. Consequently, for the third round, the gene models from the second round served as input, resulting in a new generation of gene models. Finally, a fourth annotation round was performed using MAKER in re-annotation mode. In this step, a MAKER-derived GFF3 file from the previous round was provided as input (maker_gff), allowing MAKER to retain and propagate existing evidence and predictions. In parallel, gene models were independently generated using BRAKER3 (v3), an automated genome annotation pipeline that integrates RNA-seq and protein evidence through GeneMark-ETP [57], AUGUSTUS [54, 58], and TSEBRA [59]. RNA-seq reads were aligned to the genome using HISAT2 [46], and external protein evidence was incorporated using DIAMOND-based alignments [60]. BRAKER3 was run using the same RNA-seq datasets (whole-body and antennal transcriptomes of *T. lineatum*) and the same external protein evidence sets as those used during the initial MAKER round. The resulting BRAKER3 gene predictions were supplied to MAKER as external gene models (model_gff) during the fourth annotation round. To avoid isoform redundancy in downstream comparative analyses, the parameter “alt_splice” was set to 0 in all four annotation rounds, ensuring that only a single representative gene model per locus was included in the final annotation.

Lastly, the redundancy and duplicated sequences were removed from the genome annotation generated with CD-HIT with an identity threshold of 0.98 [48], and the assembly quality and completeness assessment were performed using BUSCO (v6) with the Insecta_Odb10 database [61]. All scripts, parameter files, and detailed commands used in the genome annotation workflow are publicly available at https://github.com/lachemontes/comparativeGenomics_Tlin/blob/main/Manual/GuideYou.md.

### Functional annotation and gene orthology detection

For functional annotation, InterProScan (v5.62-94.0) was used to assign functional categories to the set of predicted protein-coding genes [62]. The analysis included the following databases: Gene3D [63], ProSite [64], Patterns [65], PANTHER [66], CDD [67], Pfam [68], Phobius [69], SUPERFAMILY [70], and TMHMM [71]. In addition, eggNOG-mapper v1.0.3 was used to assign Gene Ontology (GO) terms based on the eggNOG database [72].

Subsequently, orthology inference was performed using OrthoFinder (v2.5.2) [73]. Protein sequences from ten beetle species (*T. lineatum*, *I. typographus*, *D. ponderosae*, *H. hampei*, *T. castaneum*, *A. glabripennis*, *C. maculatus*, *L. decemlineata*, *D. virgifera*, and *Aethina tumida* Murray (Nitidulidae)) were included. Hence, apart from the fungivorous ambrosia beetle, this analysis included three related curculionid species with different dietary adaptations (two bark beetle species feeding on conifer phloem and the fructivorous coffee berry borer), as well as both generalist and specialist species from other beetle families (*Additional File 1*,* Supplementary Table 1*). *Agrilus planipennis*, *Onthophagus taurus*, and *N. vespilloides* were excluded from this analysis due to their distant phylogenetic relationships with *T. lineatum*. *Dendroctonus valens* was, on the other hand, excluded due to its small number of annotated protein-coding genes (*Additional File 1*,* Supplementary Table 1*). A data pre-processing step was applied to the external protein datasets retrieved from public databases prior to orthology inference. These reference proteomes were filtered to remove redundant sequences using CD-HIT at a 1.0 identity threshold, and proteins shorter than 100 amino acids were excluded to reduce noise from partial, fragmented gene models or low-confidence predictions.

OrthoFinder was run using gene tree inference (-M msa), multiple sequence alignment (-A mafft), and the default tree inference program. To facilitate visualization of shared and species-specific orthogroups, OrthoVenn3 [74] was used as a complementary, visualization-focused tool. The same protein datasets used for the OrthoFinder analysis were provided as input to OrthoVenn3. Orthologous clustering was performed using the OrthoFinder algorithm with default parameters (e-value threshold of 1e − 5).

To assign putative functions to orthogroups, we used InterProScan (v5.62-94.0) against the same set of databases described above. In parallel, BLAST searches against the NCBI non-redundant insect protein database (accessed April 2025) and only significant hits (e-value < 1e − 5) were retained. Orthogroup-level functional assignments were determined based on the consensus of functional annotations among member proteins, with particular emphasis on recurrent protein domains (e.g. Pfam and PANTHER) and consistent BLAST descriptions. When multiple functional descriptions were present within an orthogroup, the most frequently observed and biologically coherent annotation was used. This functional annotation of orthogroups formed the basis for downstream gene family evolution analyses with CAFE (v5) [75], enabling biological interpretation of significantly expanded and contracted gene families. Finally, GO enrichment was performed for the *T. lineatum*-specific gene families using the R package topGO (v2.54) with the weight01 algorithm and Fisher’s exact test (minimum node size = 5); p-values were additionally corrected using the Benjamini–Hochberg procedure. Pfam domain enrichment was assessed using one-sided Fisher’s exact tests, followed by a Benjamini–Hochberg correction. The background consisted of all *T. lineatum* proteins with at least one GO assignment (GO analysis) or one Pfam hit (Pfam analysis) from InterProScan.

### Gene family evolution: expansions and contractions

We used CAFE (v5) with the 13,896 orthogroups identified from the OrthoFinder analysis to investigate the evolutionary dynamics of gene families. Gene family count data from these orthogroups were reformatted to be compatible with CAFE requirements and filtered to retain gene families present in at least two species. In this analysis, we were able to estimate gene turnover rates (λ) for the 10 above-mentioned beetle species from five taxonomic families, to infer ancestral gene counts for each species, and to estimate gene gain/loss rates for each lineage of the phylogeny under a stochastic birth-and-death model. Statistical significance of gene family expansions and contractions was assessed using likelihood-based p-values implemented in CAFE, with a significance threshold of *p* < 0.05.

To improve phylogenetic inference beyond the default tree construction method (FastTree) used by OrthoFinder, we used the “SpeciesTreeAlignment” output to reconstruct a species tree with IQ-TREE (v2.4.0) [76]. The best-fit evolutionary model was selected automatically using ModelFinder [77], and was evaluated through 1,000 bootstrap iterations. The resulting phylogenetic tree was converted into an ultrametric tree using the ‘ape’ package (v3.0) in R. Divergence time estimates for *T. castaneum* and *I. typographus* were obtained from TimeTree5 to calibrate the ultrametric tree (available at https://timetree.org).

### Reconstruction of phylogenetic trees

For each gene family identified as significantly contracted or expanded, we reconstructed unrooted phylogenetic trees using the approximate maximum-likelihood method implemented in IQ-TREE (v2.4.0) [76]. The resulting phylogenetic trees (*Additional File 2*,* Supplementary Figures S2A-E*) were then visualized and annotated using iTOL (v.7) [78].

## Results and discussion

### Genome size and telomeric regions

**Fig. 1:**
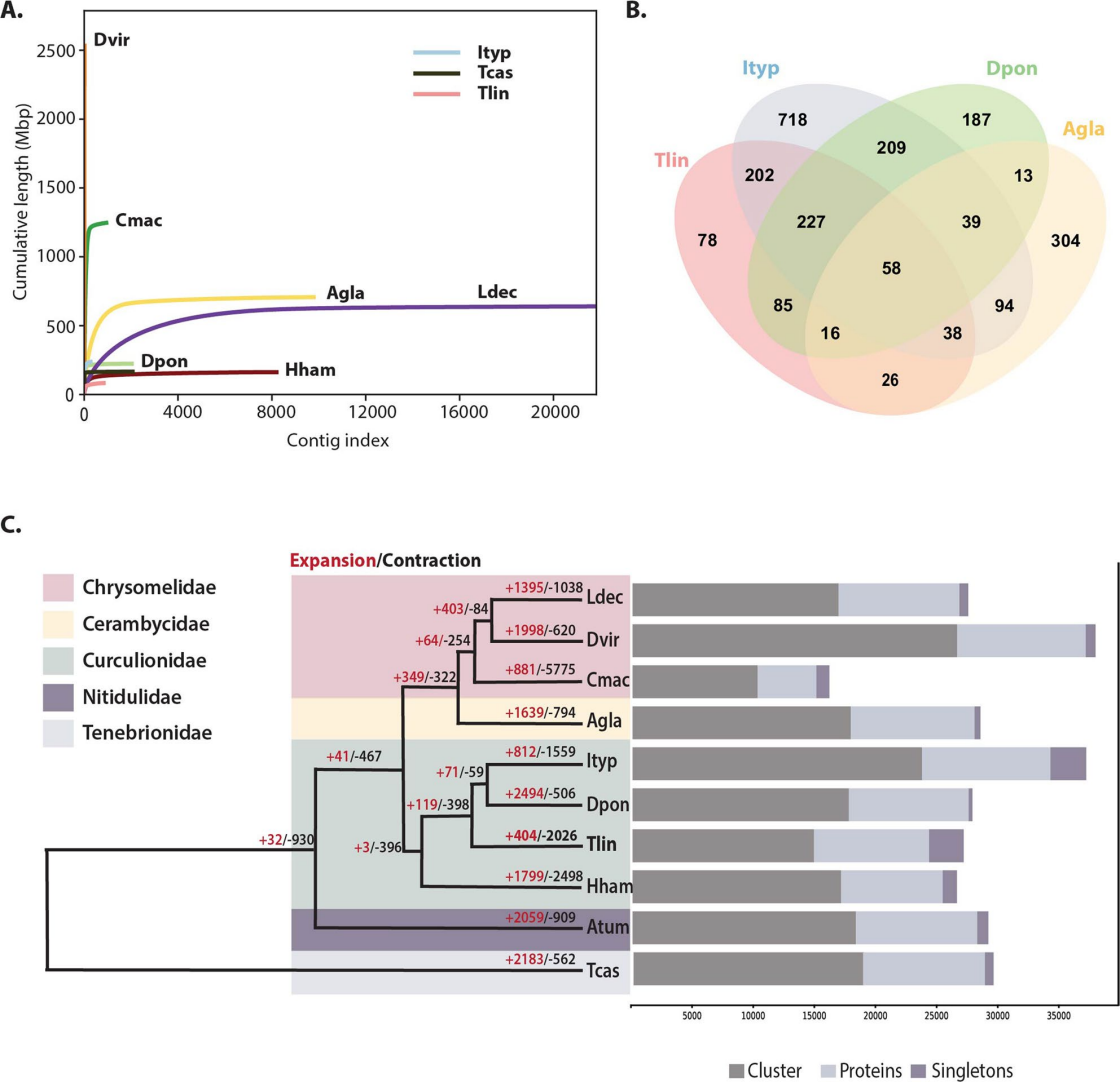
Comparative genomic features and gene family evolution across beetle species.** A** Cumulative genome assembly length (in Mbp) plotted against contig index for *Trypodendron lineatum* and 9 other coleopteran species, illustrating variation in assembly contiguity and size. The species included are *Aethina tumida* (Atum), *Anoplophora glabripennis* (Agla), *Callosobruchus maculatus* (Cmac), *Dendroctonus ponderosae* (Dpon), *Diabrotica virgifera* (Dvir), *Hypothenemus hampei* (Hham), *Ips typographus* (Ityp), and *Leptinotarsa decemlineata* (Ldec). **B** Venn diagram of orthologous groups shared between *T. lineatum* (striped ambrosia beetle), *I. typographus*, *D. ponderosae* (two bark beetles), and *A. glabripennis* (a polyphagous cerambycid wood-borer). **C** Gene family expansions and contractions inferred using CAFE for each species. Numbers indicate the total count of expanded (red) and contracted (black) gene families inferred by CAFE on each branch, including both statistically significant and non-significant changes; the subset of statistically significant changes (*p* ≤ 0.05) per species is reported in Supplementary Table 13. Species are grouped by family. The stacked bar plots on the right represent the distribution of different types of genes in the corresponding species, including single-copy, unique, and clustered genes

The k-mer analysis of raw sequencing reads produced an estimated genome size for *T. lineatum* ranging from 74.44 Mb to 83.62 Mb, which is smaller than typically observed in other beetle species (Table 1). Heterozygosity was estimated at 5.58–7.61%. In addition, the comparison of the *T. lineatum* assembly with 13 other Coleopteran genomes using QUAST (*Additional file 1*,* Supplementary Table 2*) confirmed a smaller overall assembly size. Interestingly, the assembled genome of *T. lineatum* is clearly smaller than the bark beetle genomes included in this comparison (Table 1; Fig. 1a, *Additional file 1*,* Supplementary Table 2*). Comparing genome assembly contiguity, the *T. lineatum* N_50_ value of 915 kb [36] is lower than most other beetle genome assemblies (Table 1; Fig. 1a). Telomeric repeats were not detected, which is likely due to technical limitations in assembling repetitive sequences. The small genome size of *T. lineatum* represents a notable feature within the comparative framework of coleopteran genomes analyzed here. While this pattern is consistent with a reduced repetitive content and short introns (both reported below), the extent to which genome compaction is linked to ecological specialization, symbiosis, or other evolutionary processes remains to be determined.

**Table 1:** Comparative genome assembly statistics and annotation features for *Trypodendron lineatum* and nine additional coleopteran species

Features	Tlin	Ityp	Dpon	Hham	Atum	Agla	Cmac	Ldec	Tcas	Dvir
Genome size (Mb)	83.62	236.81	223.59	162.57	259.96	706.95	1,246.71	640.05	165.92	2,533.40
Number of contigs	833	272	2,110	8,198	8	9,866	938	21,825	2,062	10
Genome assembly quality										
Contig N50 (Mb)	0.92	6.65	16.55	0.34	36.78	0.68	9.45	0.14	15.27	264.46
Contig L50	21	12	4	89	3	269	38	1172	5	5
Complete BUSCO genes (%)	99.6	99.4	98.6	97.0	99.5	99.1	99.1	93.0	99.3	97.9
Genomic features										
G + C (%)	28.23	35.21	35.82	32.32	27.93	32.84	37.99	35.47	33.86	33.05
Gene annotation										
Number of genes	15,009	23,675	17,698	17,045	18,257	17,850	10,222	16,837	18,848	26,541

Species abbreviations: Tlin (*Trypodendron lineatum*), Ityp (*Ips typographus*), Dpon (*Dendroctonus ponderosae*), Hham (*Hypothenemus hampei*), Atum (*Aethina tumida*), Agla (*Anoplophora glabripennis*), Cmac (*Callosobruchus maculatus*), Ldec (*Leptinotarsa decemlineata*), and Tcas (*Tribolium castaneum*)

### Genome annotation quality and iterative refinement

Genome annotation relied on evidence-driven approaches for both repeat identification and gene prediction. Repeat annotation using RepeatModeler found that 19.92% of the genome comprised repetitive DNA, and this fraction was masked using RepeatMasker. Of this repetitive fraction, 14.82% comprised interspersed repeats, including 3.80% retroelements (primarily long terminal repeats at 3.69%), 2.71% DNA transposons, and 0.26% rolling-circle elements. Unclassified repeats accounted for 8.31%, simple repeats for 3.99%, and low-complexity regions for 0.79%. The content of transposable elements (TEs) in the *T. lineatum* assembly is lower than the percentages reported in other coleopteran species, such as the two scolytine species *D. ponderosae* (21.12%) and *D. valens* (45.22%), as well as the flour beetle *T. castaneum* (28.90%) [79]. The TEs are major contributors to genome expansion in eukaryotes, as they replicate and insert throughout the genome, thereby increasing the overall DNA content. Large-scale analyses of > 600 insect genomes confirm a strong positive correlation (*r* ≈ 0.8–0.9) between TE-repeat abundance and genome size across orders [80]. For example, repeat content ranges from < 1% in the Antarctic midge (*Belgica antarctica*, ~ 90 Mb) [81] to ~ 10–15% in small-genome hymenopterans [82] and caddisflies (~ 150–250 Mb) [83], up to 60–70% in large-genome orthopterans like the migratory locust (*Locusta migratoria*, ~ 6.5 Gb) [84]. Compared to the scolytines *D. ponderosae* and *D. valens*, the observed reduced proportion of TEs is consistent with the smaller genome size of *T. lineatum* (*Additional File 2*,* Supplementary Table 2*), and, at a general level, it also aligns with the positive correlation between genome size and TE abundance reported in other insects [85, 86]. While a high content of TEs has been proposed to enhance environmental adaptation and invasiveness in some insects by driving genomic evolution [84, 87], the evolutionary drivers behind the reduced repetitive DNA content in the *T. lineatum* genome remain unknown. However, assembly limitations, including moderate contiguity despite high sequencing depth, suggest a potential underestimation of the total repetitive content, and completeness metrics should therefore be interpreted cautiously. In turn, the repetitive regions may have interfered with the assembly, which is a possible explanation for its moderate contiguity.

The iterative genome annotation pipeline using MAKER improved gene model quality across successive rounds, as evidenced by the progressive refinement of the AED distribution (*Additional File 2*,* Supplementary Fig. 1b*), which can range from 0 (perfect agreement with evidence) to 1 [88]. The proportion of models with strong evidence support (AED ≤ 0.3) increased from 64.0% in Round 1 (R1) to 67.0% in Round 4 (R4), indicating a modest but consistent gain in annotation precision (*Additional File 1*,* Supplementary Table 3*). Additional rounds were not performed because improvements in AED distributions started to plateau. Additionally, the completeness of the annotated genome was further assessed using BUSCO analysis based on the insecta_odb10 dataset (*n* = 1367). The analysis revealed that 95.2% of the expected genes were complete, with 93.1% identified as single-copy and 2.1% as duplicated. Only 1.9% and 2.9% of the BUSCOs were missing and fragmented, respectively, suggesting high completeness and quality of the *T. lineatum* genome annotation (*Additional File 1*,* Supplementary Table 4*). Additionally, the genome annotation generated with MAKER identified 15,009 raw genes. Subsequent redundancy filtering using CD-HIT detected 186 identical entries, resulting in a final set of 14,830 non-redundant protein sequences used for orthology inference. From these, 11,551 (76.9%) were successfully assigned at least one functional domain or Gene Ontology (GO) term using InterPro and EggNOG (*Additional File 1*,* Supplementary Tables 5 and 6*). To investigate how well our gene annotation pipeline captured the previously manually annotated *T. lineatum* chemoreceptor genes, some of which are highly divergent and often missed by automated annotation pipelines, we performed a reciprocal BLASTP comparison with the chemoreceptor catalogue reported in Biswas et al. [36]. We recovered 109 of their 140 chemoreceptor sequences (77.9%) as high-quality orthologs in our annotation, confirming broad concordance between the two annotations (*Additional File 1*,* Supplementary Table 7*). The somewhat lower number annotated in the present study is mainly explained by the collapse of recently duplicated tandem paralogs into single MAKER models.

The identified gene count (14,830) in *T. lineatum* is comparable to other coleopteran species, such as *D. ponderosae* (14,342) and *Hycleus cichorii* (13,813), and falls within the range observed in other coleopteran species [79, 89]. In addition, analysis of gene architecture (Table 2) revealed an average of 6.33 exons per gene, exceeding most species in recent comparative studies (for example, *D. virgifera* with 4.51 and *L. decemlineata* with 5.06 exons), and closely matching *C. maculatus* (6.31) and *S. oryzae* (6.35) [89]. Notably, the *T. lineatum* genome is characterized by the compactness of its genes. The average exon length (211 bp) is shorter than in all other species included in our study, and the average intron length (322 bp) is an order of magnitude smaller than in species such as *A. glabripennis* (3,214 bp) and *D. virgifera* (10,348 bp) [89]. These findings are consistent with results from a recent study that reported small introns in the manually annotated chemoreceptor genes of *T. lineatum* [36]. The combination of a high exon count and overall short introns indicates a particularly compact gene architecture in *T. lineatum*, distinguishing it from other coleopterans, including other related scolytine species. This finding opens for further investigation of the evolutionary factors underlying such a genetic structure.

**Table 2:** Gene annotation and gene architecture statistics for *Trypodendron lineatum* and *Ips typographus*

Statistic	Tlin	Ityp		
Number of genes	15,009	23,923		
Total gene length (bp)	45,826,672	132,911,182		
Longest gene (bp)	77,847	318,767		
Average gene length (bp)	3,053	5,556		
Average exon length (bp)	211	324		
Average intron length (bp)	322	957		
% of genome covered by genes	54.8	56.0		
Average exons per gene	6.33	5.00		
Average introns per gene	5.33	4.00		
Number of genes containing Pfam domains	9,555	14,145		

### Orthology inference and gene family analysis

A total of 13,896 orthogroups were identified, encompassing 93.5% of the predicted genes across all ten species in the analysis (*Additional File 1*,* Supplementary Table 8*). In *T. lineatum*, 11,986 out of the 14,830 predicted genes (80.8%) were assigned to orthogroups, while 2,844 genes remained unassigned. To assess the nature of these unassigned proteins, we compared them with the assigned set with respect to length, functional annotation coverage, and overlap with annotated transposable elements (*Additional File 1*,* Supplementary Table 9*). Unassigned proteins were significantly shorter (median: 77 amino acids) than assigned ones (385 amino acids), and a much smaller fraction carried any conserved sequence feature. These results indicate that the unassigned fraction is dominated by short, fragmentary gene models lacking conserved domain signatures, rather than constituting a reservoir of lineage-specific innovation, and is therefore unlikely to substantially affect downstream comparative analyses.

Analysis of orthogroup overlap (*Additional File 1*,* Supplementary Table 10*) revealed that *T. lineatum* shares the highest number of orthogroups with the scolytine species *H. hampei* (8,694 orthogroups), suggesting a closer relationship in terms of gene family content with this species. In contrast, *T. lineatum* shares the fewest orthogroups with the chrysomelid *C. maculatus* (3,716). In addition to shared orthogroups, OrthoFinder identified 78 *T. lineatum*-specific orthogroups, comprising 238 genes in total. Whereas the shared orthogroups were the main focus of the present study, we also addressed the *T. lineatum*-specific gene families, using GO and Pfam enrichment analyses (*Additional File 1*, *Supplementary Tables S11-S13*). Functional annotation was sparse with only 18.4% of the *T. lineatum*-specific genes carrying an InterProScan domain and 7.5% receiving a GO term. Among the few annotated genes, a significant signal for enrichment was found for digestive serine proteases, with five genes assigned both serine-type endopeptidase activity (GO:0004252) and the Trypsin Pfam domain (PF00089; FDR = 9.0 × 10⁻³). This signal complements the close-to-significant contraction of the global trypsin family detected by CAFE (see below), suggesting that while the overall trypsin repertoire has been reduced in *T. lineatum*, a small species-specific expansion also exists. Two highly enriched Pfam domains of unknown function (DUF4806 and DUF5641; FDR ≤ 5.8 × 10⁻³) represent additional candidates for future functional characterisation. Comparative visualization using OrthoVenn3 further highlighted unique orthogroup distributions across selected species (Fig. 1B).

Gene family expansion and contraction in beetles have been proposed to be linked to ecological adaptations, environmental pressures, and evolutionary history [3, 37]. These changes often involve genes associated with metabolism, sensory systems, development, digestion, and detoxification processes [90, 91]. Focusing on the orthogroups that clustered across the analyzed beetle species, 33 gene families exhibited significant size changes (*p* ≤ 0.05) in *T. lineatum* (*Additional File 1*,* Supplementary Table 14*). Of these, 16 gene families were significantly expanded, while 17 were contracted. Notably, functional annotation indicated that the contracted and expanded families encode domains associated with key key biological processes, including digestion, detoxification, host-environment interactions, and immunity (*Additional File 1*,* Supplementary Table 15*).

We found that the glycoside hydrolase family 28 (GH28, PF00295), which encodes plant cell wall degrading enzymes (PCWDEs), was significantly contracted (*p* = 0.05) in *T. lineatum*. The GH28 enzymes, especially polygalacturonases (PGs), are vital for pectin digestion, a major plant cell wall component, in many herbivorous beetles [92, 93]. These genes likely originated via horizontal gene transfer (HGT) from fungi or bacteria, then expanded through lineage-specific duplications, possibly to aid the exploitation of plant hosts [94]. This expansion supports the evolution of herbivory in the Phytophaga clade [3, 95–97]. Prior studies have reported GH28 expansions in plant-feeding species such as *D. virgifera*, *L. decemlineata*, *A. glabripennis*, and *I. typographus* [31, 92, 98]. Also, our CAFE analysis confirmed such lineage-expansions in these species. In contrast, *T. lineatum* has a much smaller GH28 repertoire, which could be due to its fungus-feeding lifestyle; although the beetles bore into xylem tissue, they do not ingest plant cell walls directly [99], potentially reducing the selective pressure to retain or expand PCWDE-related gene families. While this contraction may reflect dietary specialization, we note that gene family size changes inferred from CAFE should be interpreted cautiously, as they may also be influenced by assembly quality, annotation, or neutral lineage-specific gene turnover.

The evolution of the cytochrome P450 (PF00067) gene family is often closely associated with the ability of insects to adapt to chemically diverse environments, particularly in relation to detoxification mechanisms like host-plant interactions and insecticide resistance [100–102]. While expansions of this gene family are likely to enable insects to metabolize a broader range of foreign chemical compounds, our analysis showed that the P450 gene family is contracted in *T. lineatum* (*p* = 0.0066). Moreover, our results confirmed previous reports of the expansion of this gene family in species such as the small hive beetle (*A. tumida*) and the coffee berry borer (*H. hampei*). The small hive beetle has a large P450 gene family with 116 genes, including notable expansions in the CYP3 and CYP4 clans, which may underlie its broad metabolic capacity and potential for insecticide resistance [103]. Similarly, the coffee berry borer has an extensive P450 gene repertoire that appears specialized for detoxifying unique defensive compounds present in its host, such as chlorogenic acid derivatives and caffeine [35]. This finding suggests that a large P450 family is not a universal response to environmental challenges, but rather a reflection of the specific selective pressures at play. The evolutionary forces shaping P450s are complex, with gene family size influenced by a combination of stochastic changes and natural selection [104, 105]. For instance, even among polyphagous beetles, the evidence for P450 expansions driven by dietary shifts is limited to a small number of orthologous groups, with other detoxification families showing more pronounced enrichment [106]. Thus, the contraction observed in *T. lineatum* may be a consequence of its specialized fungus-associated lifestyle and colonization of weakened or dying trees, which reduces exposure to diverse plant secondary metabolites. However, alternative explanations such as lineage-specific gene turnover or reliance on non-P450 detoxification systems cannot be excluded.

Symbiotic interactions are fundamental to insect survival, nutrition, development, and immunity [107–109]. *T. lineatum* exemplifies this, as its obligate mutualistic relationship with *P. ferruginea* is essential for larval development [22, 26, 110]. This association likely requires immune adaptations that enable the beetle to tolerate its fungal partner in the glandular mycangia [23] while maintaining effective defenses against pathogens. Fungal symbionts are typically recognized by β-glucans, which activate pattern recognition receptors and initiate the Toll and Imd pathways, leading to NF-κB activation and the production of antimicrobial peptides [109]. Serpins are major inhibitors of immune protease cascades, including those that activate melanization and Toll signaling, and therefore play a central role in regulating the magnitude and duration of immune responses [111]. In this context, the contraction of the serpin gene family (PF00079) (*p* = 0.037) in *T. lineatum*, with only a single retained copy, may reflect lineage-specific changes in immune-related gene repertoires.

A reduced serpin repertoire could streamline immune regulation, potentially facilitating a stable relation with its obligate symbiont, as described for the pea aphid (*Acyrthosiphon pisum*) and its *Buchnera sp*. symbiont [112, 113]. We therefore hypothesize that a reduced serpin repertoire in *T. lineatum* could contribute to fine-tuning immune regulation in the presence of its obligate fungal mutualist. However, gene family size alone does not allow inference of specific immune regulatory mechanisms, and alternative explanations such as neutral lineage-specific gene turnover or annotation sensitivity may also be involved. Functional validation, including expression analyses or experimental assays would be required to address these questions. Interestingly, a similar contraction of the serpin family was observed in the bark beetle *I. typographus*, whose associated fungi aid in successful spruce colonization, both by providing nutritional benefits to the beetle and by metabolizing host defense compounds [31, 114, 115].

In contrast to the small expansion of trypsin-encoding genes among the *T. lineatum*-specific orthogroups (described above), the trypsin gene family (PF00089/IPR001254) showed a close-to-significant (*p* = 0.052) contraction in *T. lineatum* when analyzing the orthogroups shared across beetle species, similar to the serpins. Although members of this family share the conserved trypsin-like catalytic domain (IPR001254), trypsins function as digestive serine proteases, hydrolyzing dietary proteins by cleaving peptide bonds at lysine and arginine residues, and also as key regulators of immunity and development [116]. Several trypsin-like proteases function as prophenoloxidase-activating factors (PPAFs), initiating the phenoloxidase cascade and melanization, a central defense mechanism against pathogens [117]. Thus, while not strictly statistically significant, the contraction of this family in *T. lineatum* further supports the hypothesis that immune adaptability may be reduced in exchange for tolerance of its obligate fungal symbiont. In contrast, marked expansions in the trypsin gene family were identified in species such as *D. virgifera*, *A. glabripennis*, and *A. tumida*. This suggests that ecological context and symbiotic associations may contribute to shaping the evolution of immune gene families across Coleoptera.

The THAP domain (PF05485) family, which is involved in transcriptional and genomic regulation, was among the significantly (*p* = 0.0003) gene families in *T. lineatum*. The THAP domain is a type of DNA-binding domain characteristic of transcription factors, and its evolution is often linked to the activity of TEs, particularly the P-element superfamily [118, 119]. While the THAP domain family size varies across insects, notable lineage-specific expansions have been reported in species with high TE activity, such as the pea aphid (*Acyrthosiphon pisum*), which possesses hundreds of copies, likely generated through TE proliferation [120]. The significant expansion of the THAP domain family in the compact genome of *T. lineatum*, which shows a low overall content of repetitive DNA, presents an intriguing contrast. Further investigation is needed to determine the specific functions of these expanded THAP genes and their potential link to the unique genomic architecture of *T. lineatum*.

Importantly, expansions of TE-associated domain families do not necessarily follow the gradual birth–death processes assumed by CAFE [75, 121], but may instead reflect episodic bursts of transposition [122]. Consequently, inferred expansions of THAP domains should be interpreted primarily as signatures of genome dynamics rather than classical host gene family evolution, and CAFE-derived significance values for such families may be inflated due to non-independence of duplication events [121].

**Fig. 2:**
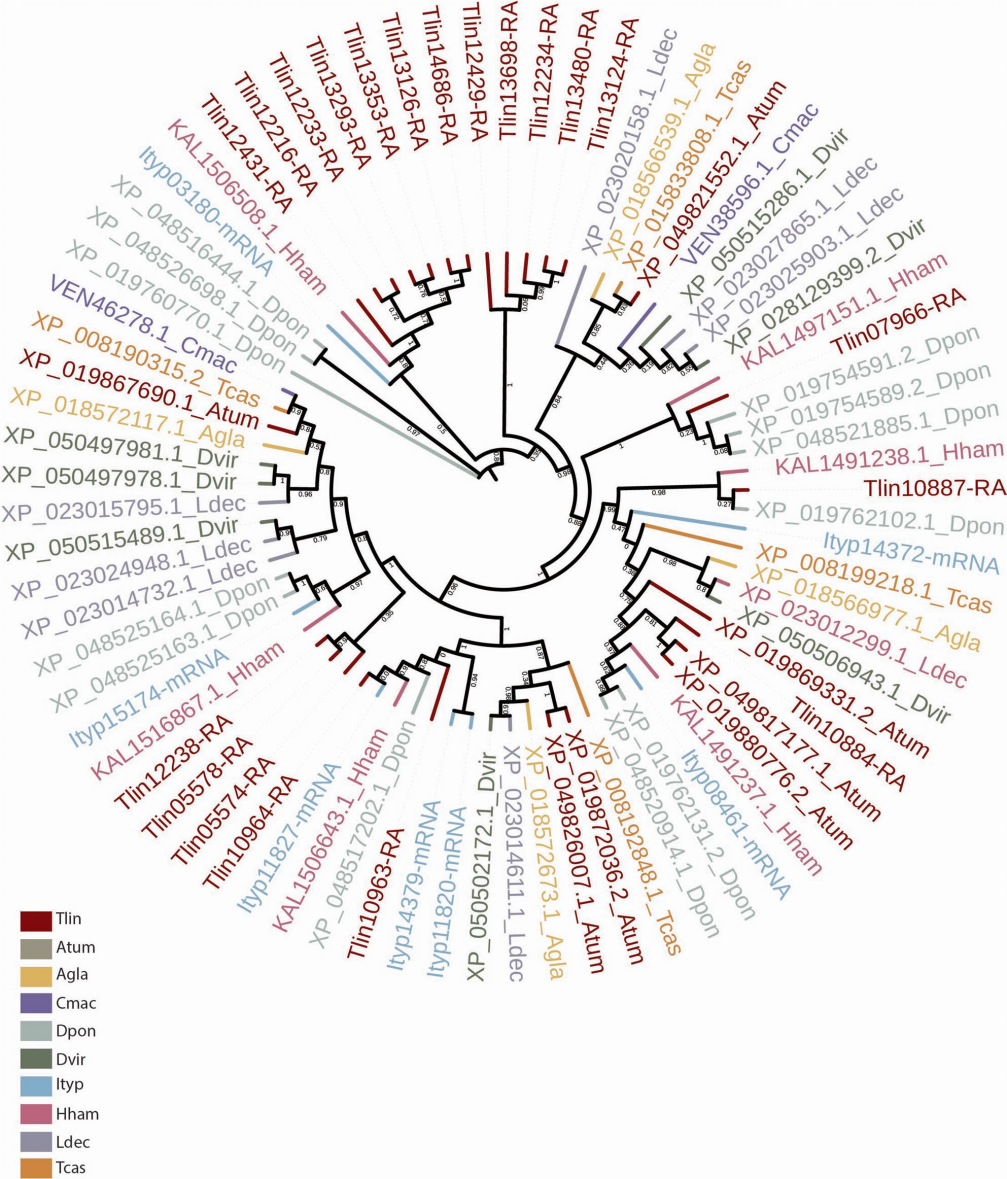
Phylogenetic analysis of orthogroup OG0000184 annotated as CD80-like C2-set immunoglobulin domain (PF08205). Phylogenetic tree of the ortholog group OG0000184, included 77 protein sequences from ten beetle species: *Trypodendron lineatum* (Tlin), *Ips typographus* (Ityp), *Dendroctonus ponderosae* (Dpon), *Hypothenemus hampei* (Hham), *Anoplophora glabripennis* (Agla), *Callosobruchus maculatus* (Cmac), *Leptinotarsa decemlineata* (Ldec), *Diabrotica virgifera* (Dvir), *Aethina tumida* (Atum) and *Tribolium castaneum* (Tcas). Support values are labelled next to the branches, which were derived from 100 bootstrap replicates. This gene family was significantly expanded in *T. lineatum*

We also found that the gene family CD80-like C2-set immunoglobulin (Ig) domain (PF08205) was significantly expanded (*p* = 0.003) in *T. lineatum* (Fig. 2). In arthropods, proteins containing this C2-set Ig domain are fundamental for cell-cell recognition, adhesion, and immune signaling, acting as receptors or co-receptors that detect pathogens and activate defense pathways [123, 124]. The expansion of this family in *T. lineatum* may indicate an evolutionary investment in the molecular mechanisms of immune recognition and communication. This is an interesting finding, given the obligate nutritional mutualism with *P. ferruginea*. Coexistence with a beneficial microorganism requires an immune system capable of distinguishing between symbionts and pathogens. We hypothesize that the expanded repertoire of CD80-like domains provides the molecular diversity necessary for immune discrimination, allowing the beetle to recognize and tolerate its fungal mutualist while maintaining the capacity to mount defenses against other invading microbes.

## Conclusions

Our comparative genomic analysis of *T. lineatum* reveals distinct genomic signatures that may reflect its specialized, obligate nutritional mutualism with its fungal symbiont, *P. ferruginea*. The results suggest that a small genome and significant changes in gene family size, particularly contractions, could be key components in the evolution of ecological specializations. The genome size, along with its reduced content of TEs and its condensed gene architecture (short introns) differ from many other beetle species. While TE proliferation often drives genomic expansion and is linked to environmental adaptation, the genomic streamlining observed in *T. lineatum* may be a specialized adaptation to its fungus-cultivating niche. This contrasts with the genomic landscapes of polyphagous or herbivorous beetles that face a wider array of environmental and host-related challenges. The contraction of the GH28 family, which encodes plant cell wall-degrading enzymes, may reflect the evolutionary shift from herbivory to fungivory. Similarly, the contraction of the cytochrome P450, serpin, and trypsin gene families suggests a potential trade-off between broad-spectrum metabolic and immune defense capabilities and the requirements of symbiotic tolerance. This reduction in immune-related gene families may represent a key mechanism enabling the beetle to maintain its relationship with its obligate fungal mutualist.

In summary, this study sheds new light on the genomic basis of symbiosis in beetles. Our findings suggest that co-evolutionary relationships with microbial mutualists may lead to a reorganization of the insect genome, characterized not by the acquisition of new defenses but by the loss or reduction of gene families that may no longer be essential for survival. This genomic “pruning” may reduce the metabolic cost of maintaining unnecessary physiological systems, while optimizing the insect for its specific, nutrient-rich niche.

## Additional file

Additional file 1: Supplementary Table 1. Summary of beetle species included in comparative genomics and orthology analyses. For each species, taxonomic family, GenBank accession number, protein sequence source, and inclusion status in ortholog analysis are indicated. Annotated proteins were downloaded in April 2025. Supplementary Table S2. Genome assembly statistics, including contig number, total assembly length, contig length metrics (N50, N90), and number of contigs greater than specific length thresholds for each beetle species used in the study. Supplementary Table S3. Cumulative distribution of Annotation Edit Distance (AED) scores across iterative MAKER rounds for *Trypodendron lineatum. *Supplementary Table S4. BUSCO assessment of genome annotation completeness for *Trypodendron lineatum* and eight additional Coleoptera species using the insecta_odb10 dataset. Supplementary Table S5. Functional annotation of *Trypodendron lineatum* gene models using InterProScan. Supplementary Table S6. Functional annotation of *Trypodendron lineatum* gene models using EggNOGmapper. Supplementary Table S7. Best BLASTp hit in the *T. lineatum* MAKER proteome for each of the 140 chemoreceptor sequences from Biswas et al. 2024 (pseudogenes excluded). Supplementary Table S8. Statistics summarizing the ortholog group analysis. Supplementary Table S9. Comparison of assigned versus unassigned *Trypodendron lineatum* proteins by OrthoFinder, in terms of length, functional annotation coverage, and transposable element overlap. Supplementary Table S10. Summary of orthogroup assignment statistics for *Trypodendron lineatum* and related beetle species. Supplemenatry Table S11. Complete Gene Ontology (GO) enrichment results for the 228 *Trypodendron lineatum*-specific genes (73 orthogroups) across the three GO ontologies: Biological Process (BP), Molecular Function (MF), and Cellular Component (CC). Supplementary Table S12. Significantly enriched GO terms (weight01 p < 0.05) for the 228 Trypodendron lineatum-specific genes, tested with topGO using the weight01 algorithm and Fisher's exact test. BP = Biological Process, MF = Molecular Function, CC = Cellular Component. FDR_BH = Benjamini–Hochberg-adjusted p-value. Supplementary Table S13. Significantly enriched Pfam protein domains in Trypodendron lineatum-specific gene families (FDR < 0.05; Fisher's exact test, one-sided, with Benjamini–Hochberg correction). Supplementary Table S14. Results of gene family expansion and contraction analysis in Trypodendron lineatum and nine additional species using CAFE. Supplementary Table S15. Functional annotation of orthogroups associated with expanded and contracted gene families in Trypodendron lineatum.

Additional file 2: Supplementary Figure 1. Genome annotation workflow and quality improvement across iterative MAKER. rounds for Trypodendron lineatum. Supplementary Figure 2A. Phylogenetic analysis of orthogroup OG0000151 annotated as Glycosyl hydrolases family 28 (PF00295). Supplementary Figure 2B. Phylogenetic analysis of orthogroup OG0000036 annotated as Cytochrome P450 (PF00067).Supplementary Figure 2C. Phylogenetic analysis of orthogroup OG0000140 annotated as Serpin (PF00079).Supplementary Figure 2D. Phylogenetic analysis of orthogroup OG0000044 annotated as Trypsin (PF00089).Supplementary Figure 2E. Phylogenetic analysis of orthogroup OG0000079 annotated as THAP domain (PF05485).

## Data Availability

The datasets supporting the conclusions of this study are included in this article and its Additional files. The genome sequence data for this study was previously deposited in the European Nucleotide Archive (ENA) at EMBL-EBI under the accession number PRJEB74033 (https://www.ebi.ac.uk/ena/browser/view/PRJEB74033). The Whole Genome Shotgun project has been deposited at GenBank under the accession GCA_055532135.1 (https://www.ncbi.nlm.nih.gov/datasets/genome/GCA_055532135.1/). All datasets generated and/or analyzed during the current study have been deposited in Figshare and are publicly available at the following repository: https://doi.org/10.6084/m9.figshare.30692345. The RNAseq reads have been deposited in the SRA database at NCBI under the accession number PRJNA1370798. In addition, all scripts, custom code, and a step-by-step reproducible workflow used for the comparative genomic analyses are openly available in the GitHub repository: https://github.com/lachemontes/comparativeGenomics_Tlin/blob/main/Manual/GuideYou.md.

## Abbreviations

AED: Annotation Edit Distance
AMP: Antimicrobial peptide
CYP: Cytochrome P450
GH28: Glycoside Hydrolase Family 28
HGT: Horizontal gene transfer
Ig: Immunoglobulin
Imd: Immune deficiency pathway
LTR: Long terminal repeat
NF-κB: Nuclear factor kappa-B
PCWDE: Plant cell wall-degrading enzyme
PG: Polygalacturonase
PPO: Prophenoloxidase
PPAF: Prophenoloxidase-activating factor
TE: Transposable element
THAP: Thanatos-associated protein domain
VOC: Volatile organic compound
