# Supplementary material for: Comparative genomics reveal signatures of ecological specialization in the striped ambrosia beetle Trypodendron lineatum
Source: BMC Genomics. 2026 Jun 16;27:549. doi: 10.1186/s12864-026-13049-3 (PMC13270666; doi:10.1186/s12864-026-13049-3)
Supplement: Supplementary file 2 — Additional file 2: Supplementary Figure 1. Genome annotation workflow and quality improvement across iterative MAKER. rounds for Trypodendron lineatum. Supplementary Figure 2A. Phylogenetic analysis of orthogroup OG0000151 annotated as Glycosyl hydrolases family 28 (PF00295). Supplementary Figure 2B. Phylogenetic analysis of orthogroup OG0000036 annotated as Cytochrome P450 (PF00067).Supplementary Figure 2C. Phylogenetic analysis of orthogroup OG0000140 annotated as Serpin (PF00079).Supplementary Figure 2D. Phylogenetic analysis of orthogroup OG0000044 annotated as Trypsin (PF00089).Supplementary Figure 2E. Phylogenetic analysis of orthogroup OG0000079 annotated as THAP domain (PF05485). [file 12864_2026_13049_MOESM2_ESM.pdf]

Additional File 2

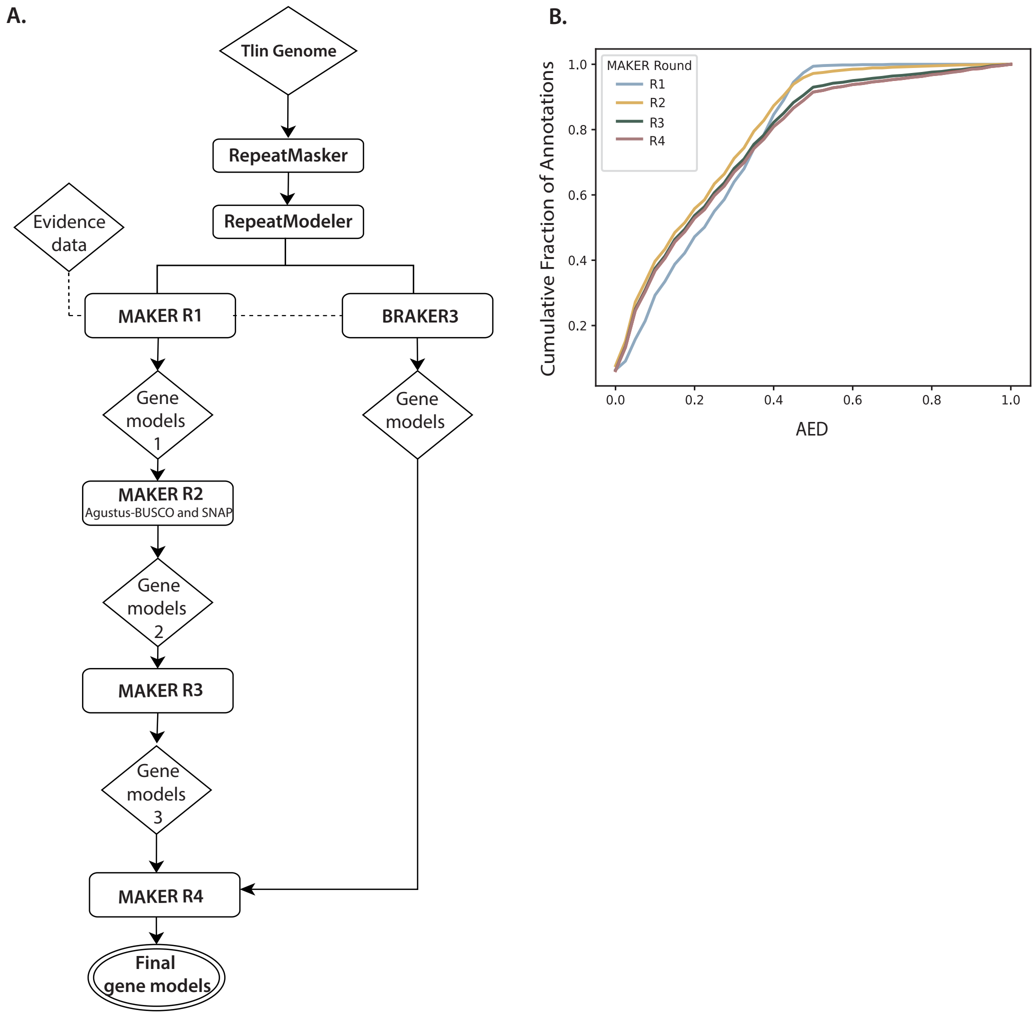

Supplementary Figure 1. (A) Workflow diagram illustrating the genome annotation strategy. The genome was first masked for repetitive elements using RepeatModeler and RepeatMasker. Gene models were predicted through four iterative rounds of the MAKER pipeline, incorporating transcriptome and protein evidence (Round 1), *ab initio* predictions using AUGUSTUS, BUSCO, and SNAP (Round 2), further refinement (Round 3), and integration with BRAKER3 predictions (Round 4). Evidence-based and *ab initio* annotations were merged to generate the final high-confidence gene models. (B) Annotation Edit Distance (AED) distribution plot across MAKER rounds. AED scores, ranging from 0 (perfect evidence support) to 1 (no support), improved consistently with each round.

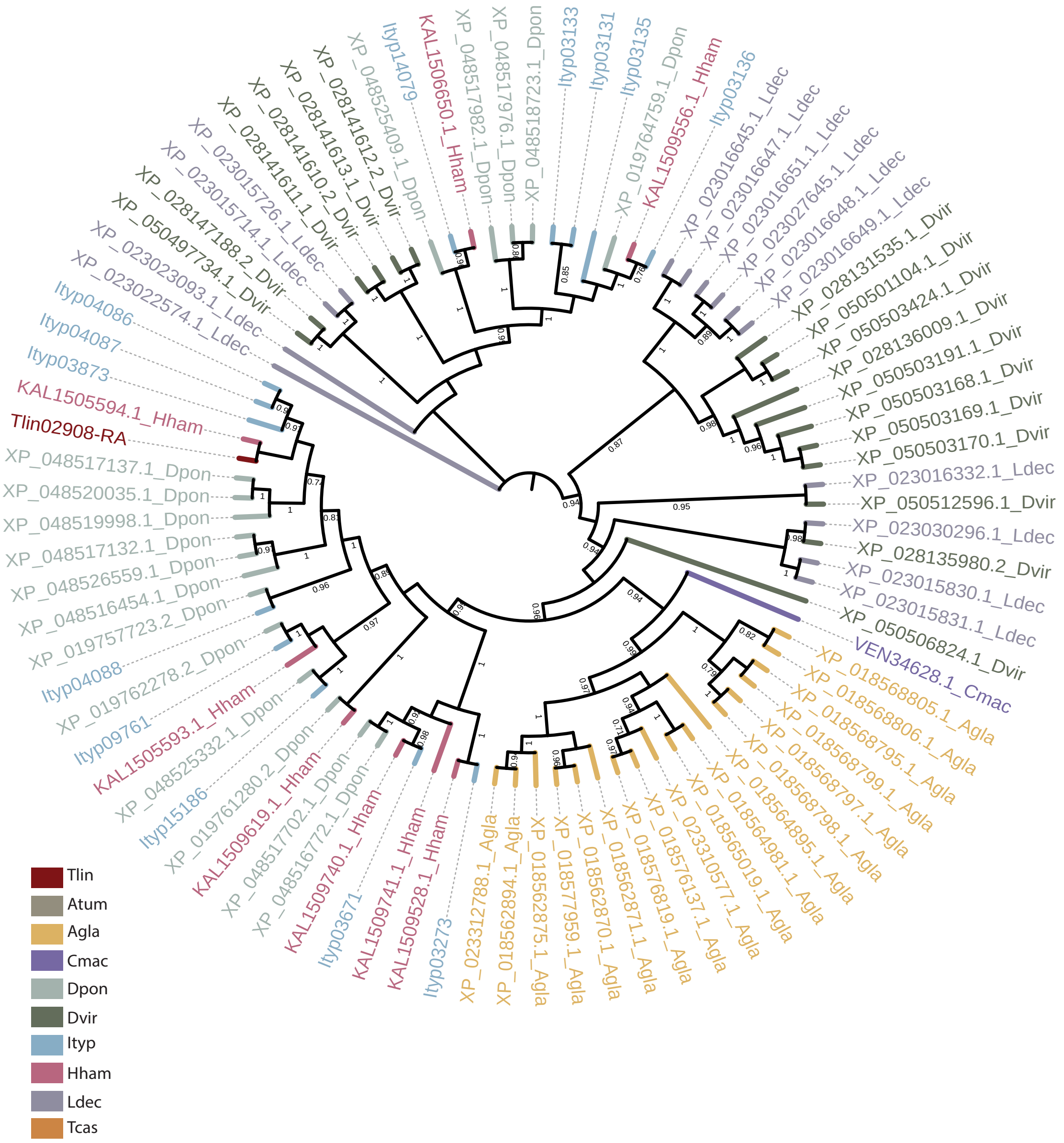

Supplementary Figure 2A. Phylogenetic analysis of orthogroup OG0000151 annotated as Glycosyl hydrolases family 28 (PF00295). The phylogenetic tree includes 195 protein sequences from ten beetle species: *Trypodendron lineatum* (Tlin), *Ips typographus* (Ityp), *Dendroctonus ponderosae* (Dpon), *Hypothenemus hampei* (Hham), *Anoplophora glabripennis* (Agla), *Callosobruchus maculatus* (Cmac), *Leptinotarsa decemlineata* (Ldec), *Diabrotica virgifera* (Dvir), *Aethina tumida* (Atum), and *Tribolium castaneum* (Tcas). Support values are labeled next to the branches, which were derived from 100 bootstrap replicates. This gene family was significantly contracted in *T. lineatum*.

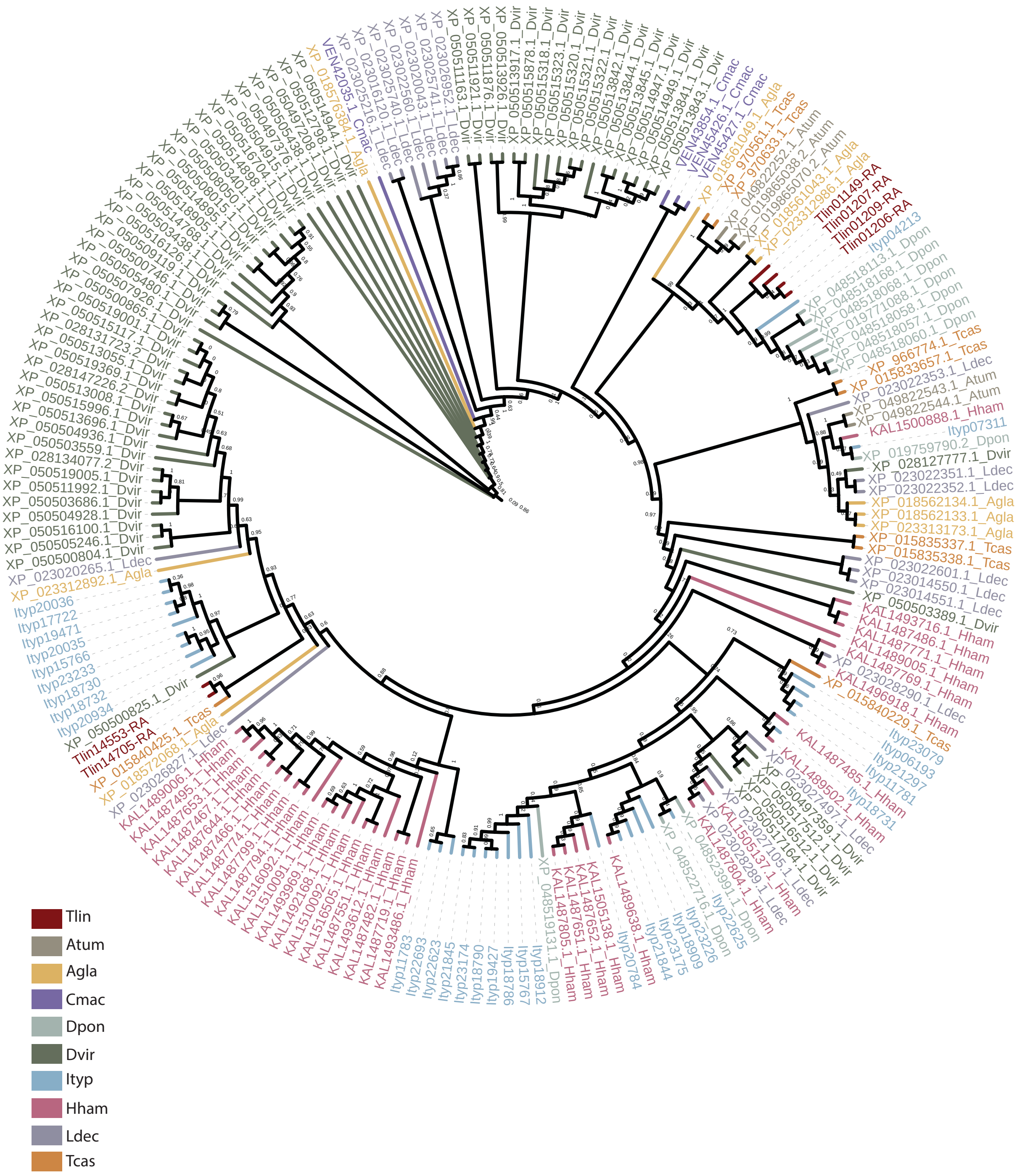

Supplementary Figure 2B. Phylogenetic analysis of orthogroup OG000036 annotated as Cytochrome P450 (PF00067). The phylogenetic tree includes 92 protein sequences from eigth beetle species: *Trypodendron lineatum* (Tlin), *Dendroctonus ponderosae* (Dpon), *Hypothenemus hampei* (Hham), *Anoplophora glabripennis* (Agla), *Leptinotarsa decemlineata* (Ldec), *Diabrotica virgifera* (Dvir), *Aethina tumida* (Atum) and *Tribolium castaneum* (Tcas). Support values are labeled next to the branches, which were derived from 100 bootstrap replicates. This gene family was found to be significantly contracted in *T. lineatum*.

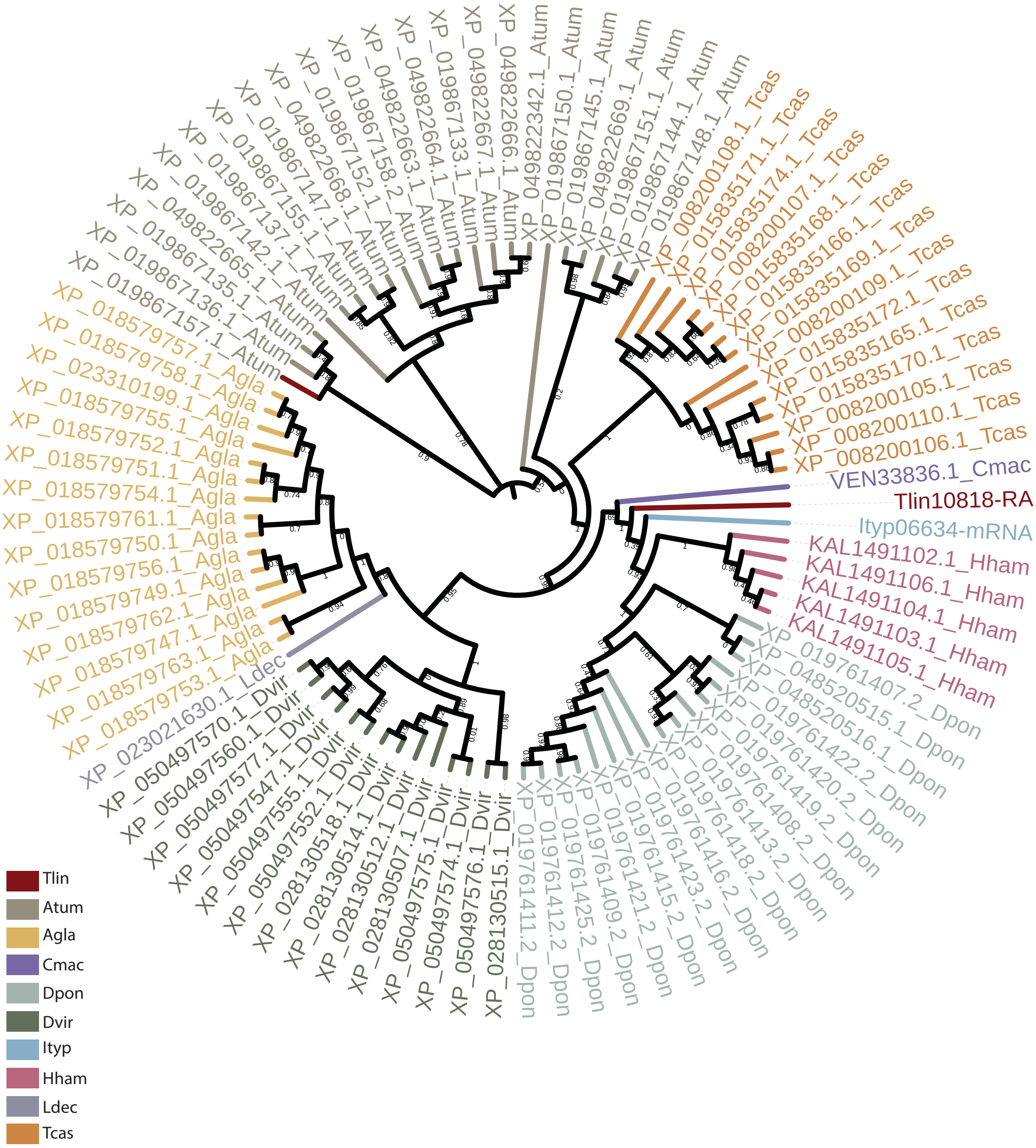

Supplementary Figure 2C. Phylogenetic analysis of orthogroup OG0000140 annotated as Serpin (PF00079). The phylogenetic tree includes 92 protein sequences from eight beetle species: *Trypodendron lineatum* (Tlin), *Dendroctonus ponderosae* (Dpon), *Hypothenemus hampei* (Hham), *Anoplophora glabripennis* (Agla), *Leptinotarsa decemlineata* (Ldec), *Diabrotica virgifera* (Dvir), *Aethina tumida* (Atum), and *Tribolium castaneum* (Tcas). Support values are labeled next to the branches, which were derived from 100 bootstrap replicates. This gene family was found to be significantly contracted in *T. lineatum*.

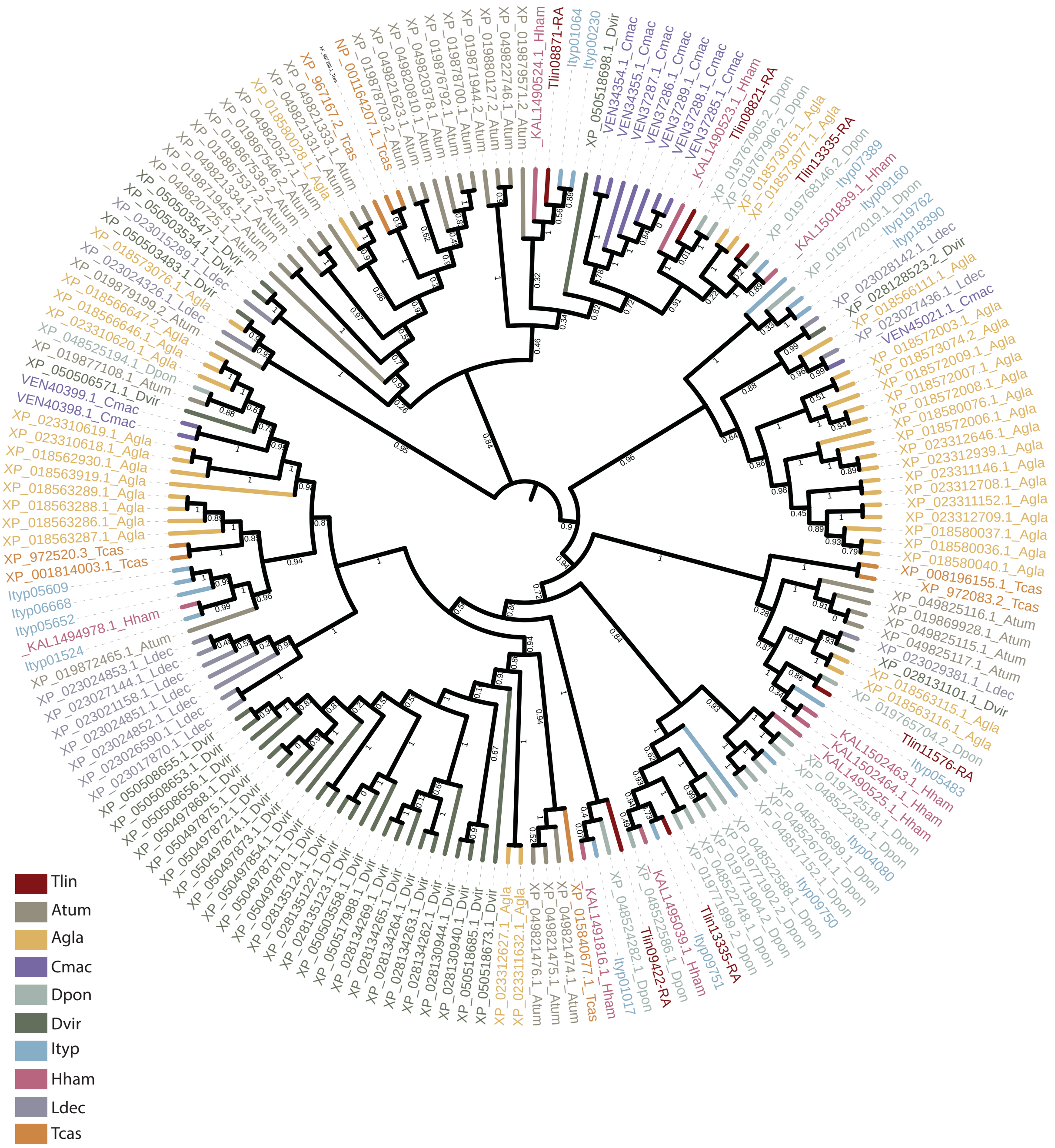

Supplementary Figure 2D. Phylogenetic analysis of orthogroup OG0000044 annotated as Trypsin (PF00089). The phylogenetic tree includes 175 protein sequences from nine beetle species: *Trypodendron lineatum* (Tlin), *Ips typographus* (Ityp), *Dendroctonus ponderosae* (Dpon), *Hypothenemus hampei* (Hham), *Anoplophora glabripennis* (Agla), *Callosobruchus maculatus* (Cmac), *Leptinotarsa decemlineata* (Ldec), *Diabrotica virgifera* (Dvir), *Aethina tumida* (Atum) and *Tribolium castaneum* (Tcas). Support values are labeled next to the branches, which were derived from 100 bootstrap replicates. This gene family was found to be significantly contracted in *T. lineatum*.

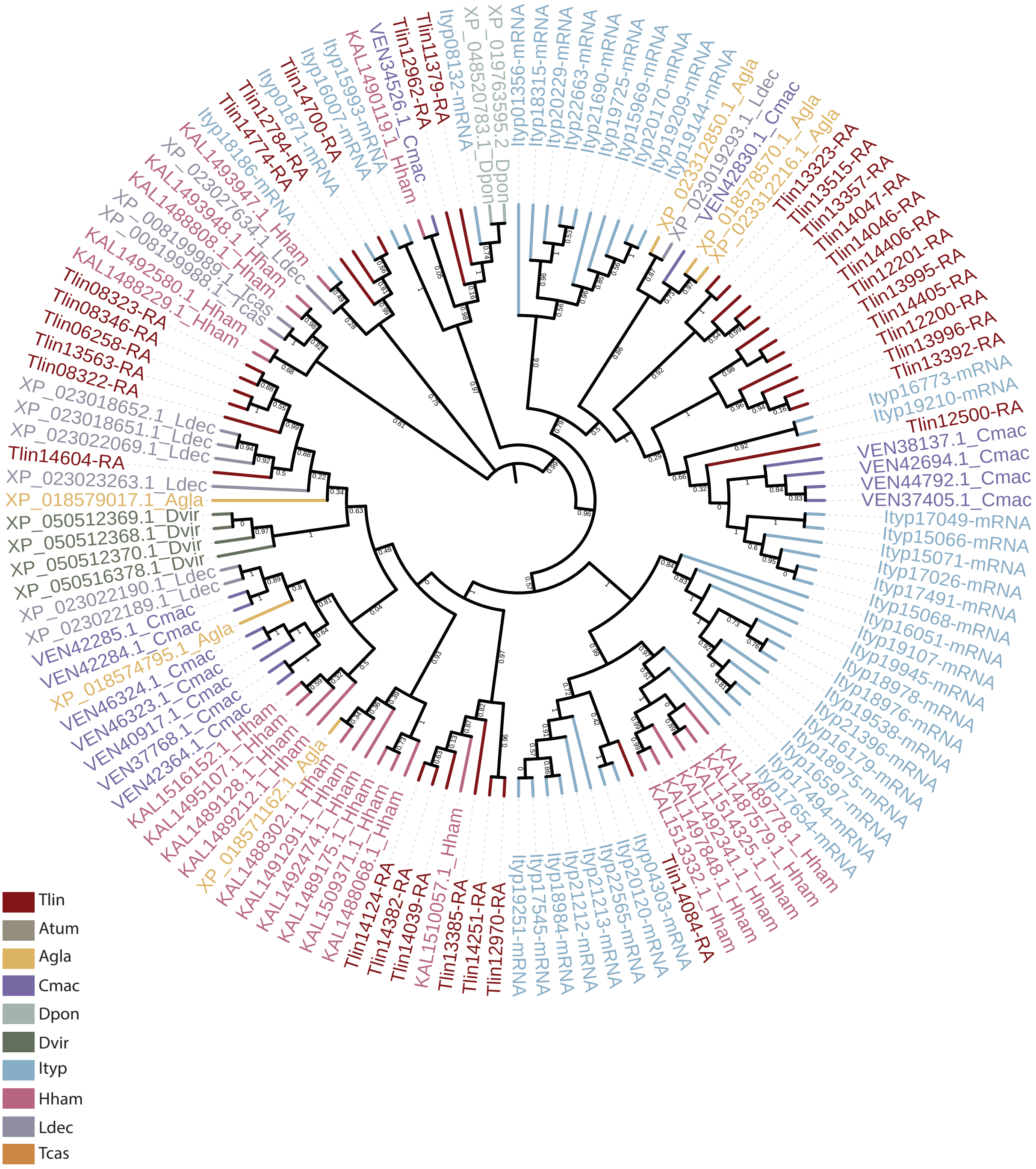

Figure S2E. Phylogenetic analysis of orthogroup OG0000079 annotated as THAP domain (PF05485). The phylogenetic tree includes 132 protein sequences from nine beetle species: *Trypodendron lineatum* (Tlin), *Ips typographus* (Ityp), *Dendroctonus ponderosae* (Dpon), *Hypothenemus hampei* (Hham), *Anoplophora* glabripennis (Agla), *Callosobruchus maculatus* (Cmac), *Leptinotarsa decemlineata* (Ldec), *Diabrotica virgifera* (Dvir), and *Tribolium castaneum* (Tcas). Support values are labeled next to the branches, which were derived from 100 bootstrap replicates. This gene family was significantly expanded in *T. lineatum*.
